# Resident worklife and wellness through the late phase of the pandemic: a mixed methods national survey study

**DOI:** 10.1186/s12909-024-05480-5

**Published:** 2024-05-02

**Authors:** Mark Linzer, Sanjoyita Mallick, Purva Shah, Anne Becker, Nancy Nankivil, Sara Poplau, Shivani K. Patel, Caitlin Nosal, Christine A. Sinsky, Elizabeth Goelz, Martin Stillman, Michaella Alexandrou, Erin E. Sullivan, Roger Brown

**Affiliations:** 1 Institute for Professional Worklife, Hennepin Healthcare, 701 Park Avenue, Minneapolis, MN 55415 USA; 2 Department of Medicine, Hennepin Healthcare, 701 Park Avenue, Minneapolis, MN 55415 USA; 3 American Medical Association, 330 N. Wabash Avenue, Chicago, IL 60611 USA; 4 Minneapolis Heart Institute, 800 East 28th St., Minneapolis, MN 55407 USA; 5 Sawyer School of Business, Harvard Medical School and Suffolk University, 73 Tremont St, Boston, MA 02108 USA; 6 School of Nursing, University of Wisconsin, 701 Highland Avenue, Madison, WI 53705 USA

**Keywords:** Burnout, Stress, Sleep impairment, Gender differences, Residency training

## Abstract

**Background:**

System contributors to resident burnout and well-being have been under-studied. We sought to determine factors associated with resident burnout and identify at risk groups.

**Methods:**

We performed a US national survey between July 15 2022 and April 21, 2023 of residents in 36 specialties in 14 institutions, using the validated Mini ReZ survey with three 5 item subscales: 1) supportive workplace, 2) work pace/electronic medical record (EMR) stress, and 3) residency-specific factors (sleep, peer support, recognition by program, interruptions and staff relationships). Multilevel regressions and thematic analysis of 497 comments determined factors related to burnout.

**Results:**

Of 1118 respondents (approximate median response rate 32%), 48% were female, 57% White, 21% Asian, 6% LatinX and 4% Black, with 25% PGY 1 s, 25% PGY 2 s, and 22% PGY 3 s. Programs included internal medicine (15.1%) and family medicine (11.3%) among 36 specialties. Burnout (found in 42%) was higher in females (51% vs 30% in males, *p* = 0.001) and PGY 2’s (48% vs 35% in PGY-1 s, *p* = 0.029). Challenges included chaotic environments (41%) and sleep impairment (32%); favorable aspects included teamwork (94%), peer support (93%), staff support (87%) and program recognition (68%). Worklife subscales were consistently lower in females while PGY-2’s reported the least supportive work environments. Worklife challenges relating to burnout included sleep impairment (adjusted Odds Ratio (aOR) 2.82 (95% CIs 1.94, 4.19), absolute risk difference (ARD) in burnout 15.9%), poor work control (aOR 2.25 (1.42, 3.58), ARD 12.2%) and chaos (aOR 1.73 (1.22, 2.47), ARD 7.9%); program recognition was related to lower burnout (aOR 0.520 (0.356, 0.760), ARD 9.3%). These variables explained 55% of burnout variance. Qualitative data confirmed sleep impairment, lack of schedule control, excess EMR and patient volume as stressors.

**Conclusions:**

These data provide a nomenclature and systematic method for addressing well-being during residency. Work conditions for females and PGY 2’s may merit attention first.

**Supplementary Information:**

The online version contains supplementary material available at 10.1186/s12909-024-05480-5.

## Introduction

The pandemic produced upheavals in worklife for practicing clinicians and staff. While national studies have assessed worklife in practicing physicians [1–4] and staff [5], fewer have addressed resident worklife [6]. Much of the literature is from the 2000’s and 2010’s [7–9], and most studies employ data from small numbers of residents and programs. Burnout prevalence rates vary considerably, from 35 to 76% [7–9]. Yet little is available to determine how residents traversed the pandemic, and how to prepare for future surges in stress.

We reviewed recent data (July 2022 to April 2023) from residency programs surveyed by the American Medical Association (AMA) using the Mini ReZ, a validated measure [10] derived from the Mini Z [11] assessing burnout with a single item validated against the Maslach Burnout Inventory (MBI) emotional exhaustion (EE) scale [12], and several items addressing known components of burnout [13, 14], as well as 5 items derived from Trockel [15] defining work conditions related to resident burnout (interruptions, sleep impairment, support staff relationships, recognition by program and peer support). Study objectives were to determine 1) burnout prevalence, 2) program characteristics associated with favorable burnout rates, 3) gender differences in resident burnout (found previously in faculty and practicing clinicians), and 4) differences in work conditions by Post Graduate Year (PGY), anticipating that PGY 1 year would be most stressful. We used qualitative analysis in a “complementarity” manner to enhance findings from quantitative scales, focusing on remediable correlates of burnout.

## Methods

### Sample

In 2017, the AMA began surveying residencies using the Mini Z for residents (Mini ReZ). For this paper we focus on 14 institutions and 1118 residents surveyed from July 15, 2022 through April 21, 2023. Residents trained in varied specialties (see Supplemental Table 1A), with the most in internal medicine, family medicine and emergency medicine. Response rates, determined by institution, allowed calculation of an overall median rate.

**Table 1:** Sample demographics (*n* = 1118 residents, July 2022 – April 2023) with baseline worklife summary scores and subscale scores

	**N (%)**		
**Gender**			
Male	507 (46)		
Female	529 (48)		
PNTI, gender	66 (6)		
**Race/Ethnicity**			
White	598 (57)		
Latinx	61 (6)		
Black	47 (4)		
Asian	220 (21)		
Other	14 (1)		
PNTI, race/ethnicity	119 (11)		
**Years of training**			
PGY 1	266 (25)		
PGY 2	269 (25)		
PGY 3	234 (22)		
PGY 4	130 (12)		
PGY 5	50 (5)		
Fellow	124 (12)		
**Summary and subscale scores**	**Mean**	**Potential range**	**% of total possible**^a^
Worklife (summary) score	51.0	15–75	68%
Supportive work environment (subscale 1)	18.9	5–25	75.6%
Work pace/EMR stress (subscale 2)	14.4	5–25	57.6%
Sleep/program support (subscale 3)	17.7	5–25	70.8%

*Abbreviations*: *EMR*  Electronic Medical Record, *PGY*  Post Graduate Year, *PNTI*  Prefer not to indicate gender or race

^a^target for all 4 scales, > 80% of total possible score = joyous workplace. Scoring in Appendix

### Study design

Residents were surveyed anonymously, typically once per year. Organizations performed their own surveys, and results were aggregated in the affiliated Data Lab.

### Measure

The Mini ReZ (Supplemental Fig. 1A) uses the core Mini Z 10 item structure, assessing outcomes (satisfaction, stress and burnout), and work conditions (work control, chaotic environments, teamwork, values alignment and electronic medical record (EMR) experience) using 5-point scales [11]. Five items were added to reflect findings from Trockel [16] of domains critical to resident wellness (interruptions, sleep impairment, support staff relationships, program recognition and peer support). Questions were aligned from low to high (high score = positive attribute). Items were dichotomized with the top 2 or 3 choices scored as, e.g., “good control”, “no chaos”, or “efficient teamwork”. Details on subscales, scoring [17] and validation [18, 19] are in the Technical Appendix. In brief, a summary score of 75 (5 × 15, range 15–75, > 80% = a “joyous workplace”) is created, consisting of three 5 item subscales: 1) supportive work environment (range 5–25, target = 20 or higher), 2) work pace/EMR stress (range 5–25, target of 20 or higher) and 3) resident specific factors (sleep, interruptions, peer and staff support, and program recognition, range 5–25, target 20 or higher).

**Fig. 1:**
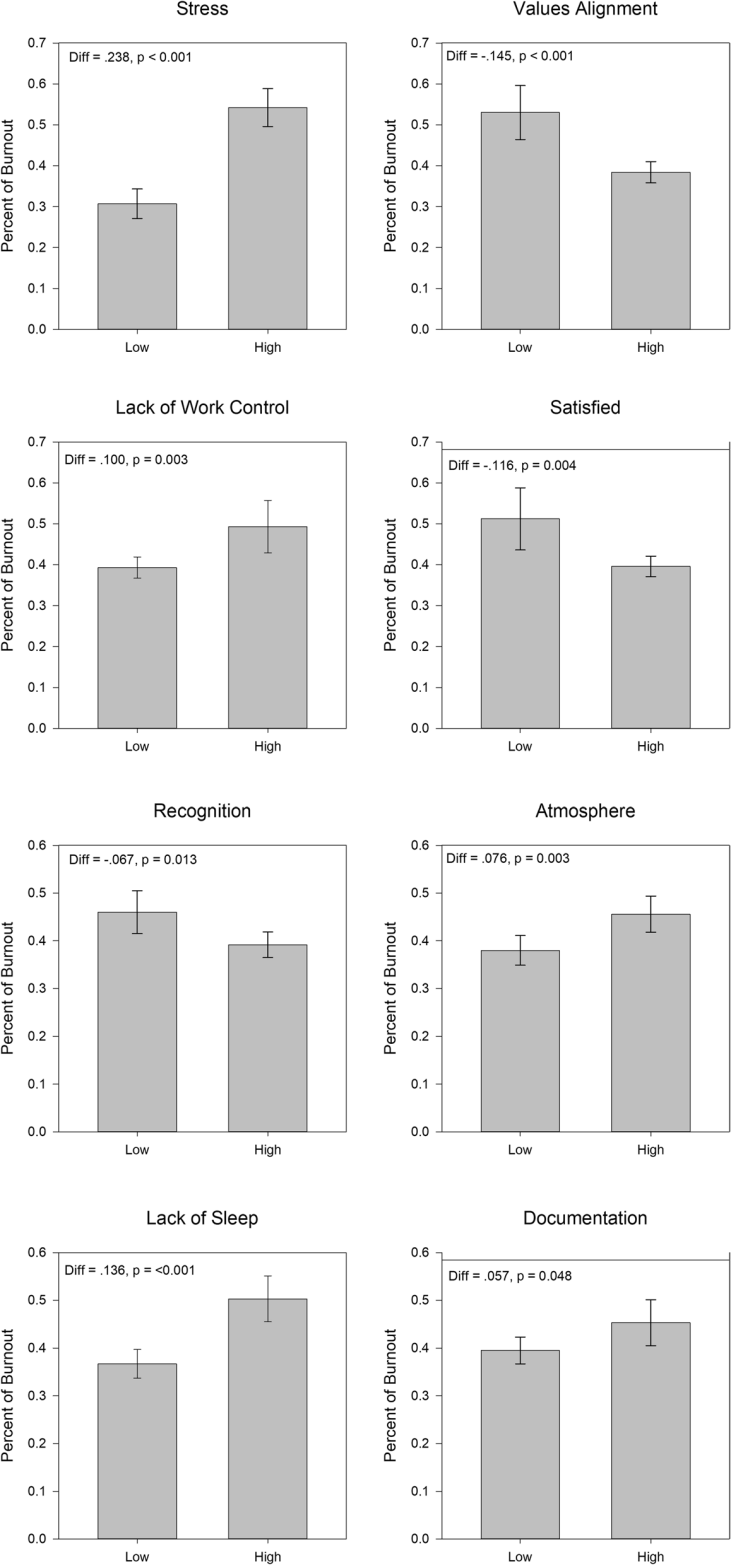
Burnout by predictor variables (satisfaction, chaos (work atmosphere), values alignment, recognition by program, lack of work control, stress, documentation time pressure and sleep impairment) in 1118 residents in national Mini ReZ survey July 2022 to April 2023. “High” = variable present (e.g. high satisfaction, top two scores), “low” means variable had lower scores

### Quantitative analysis

Bivariate comparisons were performed using Chi square, t tests and Fisher’s exact test, correcting for multiple comparisons with the FDR (False Discovery Rate). Higher scores were collapsed into binary variables, noting “presence” of a variable (e.g. values alignment) vs absence. Multivariate regressions determined remediable correlates of burnout. A p value of < 0.05 was considered statistically significant. Absolute differences of 5–10% in burnout rates or in prevalence of work conditions were considered clinically meaningful, correlating with an Effect Size (ES) of 0.1 to 0.2. Forest plots assessed standardized mean differences, with ESs representing important differences between genders and PGY years (1, 2, 3 or 4/5/Fellow).

### Qualitative analysis

 [20] Thematic analysis assessed additional factors related to burnout. Responses to the open-ended question, “Tell us more about your current stressors and ideas you have for minimizing them,” were analyzed using an inductive, thematic approach. First, comments were reviewed to identify emerging and recurrent themes. Comments were then thematically indexed and coded using NVivo 12. Co-authors reviewed results and reached consensus on how qualitative data contextualized quantitative findings.

Qualitative data which enhanced interpretation of quantitative data were merged with quantitative findings in line with theoretical constructs of the Job-Demands Resources (JD-R) [21] and Demand-Control Models of job stress [22], as well as healthcare-related application of these models in the MEMO study (Minimizing Error Maximizing Outcome) [13] and Healthy Work Place trial [23], to create a conceptual model of worklife and well-being in residents.

The Hennepin Healthcare Institutional Review Board (IRB) determined this work was exempt from human subjects research requirements.

## Results

### Demographics

There were 1118 respondents in 14 institutions (with 36 program types listed in Supplemental Table 1A). Median response rate was approximately 32% (25th percentile 19%, 75th percentile 94%, interquartile range 75%). Respondents were located in the Midwest (*N* = 179, 16.0%), Northeast (*N* = 530, 47.4%), Southern (*N* = 244, 21.8%) and Western (*N* = 165, 14.8%) US regions. Of respondents (Table 1), 507 (46%) were male, 529 (48%) female, and 66 (6%) preferred not to identify gender (PNTI-g); 598 (57%) identified as White, 220 (21%) Asian, 61 (6%) Latinx, 47 (4%) Black, and 119 (11%) preferred not to identify race or ethnicity (PNTI-r). For year of training, 25% were PGY 1’s, 25% PGY 2’s, 22% PGY 3’s, with the remainder PGY 4’s, 5’s and Fellows.

### Outcomes

#### Summary score and subscales (Table 1)

The summary score and 3 subscale scores were all less than target (80% of possible top score). Three were > 65% of total possible score, while one scale (“work pace/EMR stress”) was moderately lower at 58% of possible.

For *individual worklife item prevalence*, program satisfaction was high in 83% of residents (Tables 2 and 3). Burnout was present in 42%, higher in females (51%) and highest in those preferring not to identify gender (56%) or race (57%). Values alignment with leaders, a correlate of lower burnout [13] in practicing physicians, was high in 78% of residents, while teamwork, related to lower burnout in clinical practice [24], was rated highly by 94%. Lack of work control, a factor associated with burnout during the pandemic [5] and in prior years [25] in clinicians, was poor or marginal in 22%, while high stress, an antecedent of burnout, was noted by 44%. High home EMR use was noted by 34%, and chaotic environments, another burnout correlate [26] in practicing physicians, were described by 41% of residents.

**Table 2:** Characteristics of resident work life, overall and stratified by gender in 1118 residents in national mini ReZ survey, July 2022 to April 2023

	Overall %	In males	In females	DIFF (M-F)	FDR *p*-value
Subscale 1: Supportive workplace					
Satisfaction	83%	88%	81%	7%	0.003
Burnout	42%	30%	51%	-21%	0.001
Values alignment	78%	84%	75%	8%	0.392
Teamwork	94%	97%	94%	3%	0.001
Work control (poor/marginal)	22%	16%	26%	-10%	0.001
Subscale 2: Work pace and EMR issues					
Stress (high)	44%	33%	52%	-19%	0.001
Home EMR (high)	34%	29%	37%	-7%	0.019
Time pressure documenting	29%	25%	30%	-4%	0.127
Chaos	41%	35%	45%	-10%	0.001
Frustration with EMR	47%	43%	48%	-5%	0.101
Subscale 3: Residency-specific experiences					
Interruptions	27%	23%	28%	-5%	0.079
Lack of sleep	32%	28%	35%	-7%	0.019
Positive staff relationships	87%	92%	84%	8%	0.001
Peer support	93%	95%	91%	4%	0.019
Program recognition	68%	73%	64%	9%	0.002
Summary and subscale scores					
Summary and subscale scores					*p* values
Summary scores (mean)	51.0	53.6	49.4	4.25	0.001
Supportive environment	18.9	19.9	18.3	1.63	0.001
Work pace/EMR stress	14.4	15.3	13.9	1.34	0.001
Sleep/program support	17.7	18.5	17.2	1.28	0.001

*EMR* Electronic Medical Record, *FDR* False Discovery Rate (correction for multiple comparisons), *PNTI* Prefer not to indicate gender or race

**Table 3:** Characteristics of resident work life, overall and stratified by year in training in 1118 residents in national mini ReZ survey, July 2022 to April 2023

	Overall %	In PGY1	In PGY2	In PGY3	DIFF (PGY1—PGY2)	FDR *p*-value	DIFF (PGY1 PGY3)	FDR *p*-value
Subscale 1: Supportive workplace								
Satisfaction	83%	88%	82%	81%	6%	0.168	6%	0.163
Burnout	42%	35%	48%	47%	-13%	0.029	-11%	0.083
Values alignment	78%	82%	77%	76%	5%	0.311	5%	0.311
Teamwork	94%	98%	91%	94%	7%	0.024	4%	0.163
Work control (poor/marginal)	22%	20%	27%	22%	-8%	0.163	-3%	0.675
Subscale 2: Work pace and EMR issues								
Stress (high)	44%	44%	49%	46%	-4%	0.498	-1%	0.957
Home EMR (high)	34%	31%	40%	36%	-9%	0.163	-5%	0.440
Time pressure documenting	29%	27%	33%	26%	-6%	0.311	1%	0.957
Chaos	41%	41%	46%	41%	-5%	0.400	0%	0.973
Frustration with EMR	47%	46%	46%	44%	0%	0.973	2%	0.869
Subscale 3: Residency-specific experiences								
Interruptions	27%	29%	30%	22%	-1%	0.957	7%	0.179
Lack of Sleep	32%	38%	38%	28%	0%	0.973	10%	0.125
Positive Staff relationships	87%	85%	85%	88%	0%	0.973	-4%	0.385
Peer support	93%	94%	93%	93%	1%	0.957	0%	0.957
Program recognition	68%	70%	64%	69%	6%	0.311	1%	0.957
Summary and subscale scores								
Summary scores (mean)	51.0	51.3	49.1	51.7	2.2	0.083	-0.40	0.163
Supportive environment	18.9	19.4	18.3	18.7	1.1	0.011	0.64	0.556
Work pace/EMR stress	14.4	14.4	13.7	14.7	0.6	0.163	-0.32	0.163
Sleep/program support	17.7	17.5	17.1	18.2	0.4	0.385	-0.70	0.916

*EMR* Electronic Medical Record, *FDR* False Discovery Rate (correction for multiple comparisons), *PNTI* Prefer not to indicate gender or race

PNTI-gender group (*n* = 66) had challenging scores for sleep impairment (33%), program recognition (62%), excess EMR at home (39%), chaos (55%), low work control (35%), and burnout (56%)

Of resident-specific domains, sleep impairment, a burnout correlate [16], was noted by 32%, positive relationships with support staff were described by 87%, peer support (typically felt to refer to support by resident peers) was noted by 93%, while recognition by program was noted by 68%. Burnout—work environment graphs (Fig. 1) show lower burnout with high satisfaction, values alignment and recognition by program, and higher burnout in the presence of stress, chaos (work atmosphere), lack of work control, documentation (EMR) pressures and sleep impairment (all p’s < 0.05).

#### Gender differences

Burnout was higher among females vs males (51% vs 30%, *p* = 0.001). Other variables were consistently poorer in females, including poor work control (26% poor or marginal control in females vs 16% in males, *p* = 0.001), high stress (52% highly stressed in females vs 33% of males, *p* = 0.001), high home EMR use (in 37% of females vs 29% of males, *p* = 0.019), chaotic workplaces (in 45% of females vs 35% in males, *p* = 0.001), and sleep impairment (in 35% of females vs 28% of males, *p* = 0.019). Summary scores (49.4 (out of 75) in females vs 53.6 in males, absolute difference 4.25, adjusted *p* = 0.001), and all 3 subscales (supportive environment, work pace/EMR stress, and resident-specific items) were significantly lower in females (adjusted *p* values = 0.001).

#### Program year

Differences were also seen by program year. High satisfaction was most often seen (88% of the time) in PGY 1’s vs 82% or lower in PGY 2’s and 3’s; burnout was less often seen in PGY 1’s at 35% of the time (vs 48% in PGY 2’s (*p* = 0.029) and 47% in PGY 3’s, *p* = 0.083). Efficient teamwork was endorsed by 98% of PGY 1’s, vs 91% in PGY 2’s (*p* = 0.024) and 94% in PGY 3’s (*p* = 0.163). The most frequent endorsement of excessive home EMR time was by PGY 2’s at 40%. Sleep impairment was noted equally as often by PGY 2’s as PGY 1’s (38%). Recognition by one’s program was noted least often by PGY 2’s (64%), although the difference with PGY 1’s (70% recognized) was not statistically significant. The supportive work environment subscale (18.3 vs 19.4) was lower in PGY 2’s vs PGY 1’s (adjusted *p* = 0.011).

The *Forest plot in *Supplemental Fig. 2A assesses subscale scores by gender and year, including PGY 1’s, 2’s, 3’s and 4’s/5’s/Fellows as a final category. Fellows had favorable findings, and SMDs (standardized mean differences, or Effect Sizes) showed prominent differences for males vs females (small to moderate ESs favoring males) for all 3 subscales. Greater challenges were seen for those not identifying gender.

#### Regression analyses assessing potential components of burnout

In multivariate regressions controlling for gender and year of training (Table 4), favorable worklife aspects included program satisfaction (adjusted Odds Ratio (aOR) in association with burnout 0.415, *p* = 0.002) and recognition by program (aOR 0.606, *p* = 0.012), while challenging factors included stress (aOR 4.47 for greater burnout, *p* < 0.001), sleep impairment (aOR 2.58, *p* < 0.001), lack of work control (aOR 2.04, *p* = 0.003) and chaos (aOR 1.69, *p* = 0.004). The full regression model (Table 4 and Supplemental Table 2A) explained 55% of variance in burnout.

**Table 4:** Multilevel regressions of burnout-related work conditions in 1118 residents in the national Mini ReZ survey study July 2022 to April 2023

		Resident Burnout *N* = 1,118 Organizations = 12								
Mini-Rez Items	AOR	95% CI		*p*-value	ARR	95% CI		ARD	95% CI	
Satisfaction	.415*	.240	.719	0.002	0.752	0.632	0.894	-0.12	-0.21	-0.04
Values alignment	.428	.268	.682	0.000	0.754	0.647	0.880	-0.12	-0.19	-0.05
Teamwork efficiency	.552	.203	1.502	0.245	0.829	0.614	1.12	-0.08	-0.22	0.06
Poor work control	2.035	1.272	3.257	0.003	1.26	1.08	1.48	0.10	0.03	0.17
High stress	4.472	3.110	6.430	0.000	1.82	1.54	2.15	0.24	0.18	0.31
EMR use outside of work	.956	.636	1.437	0.832	0.98	0.86	1.12	-0.005	-0.05	0.04
Time pressure	1.550	1.004	2.391	0.047	1.15	1.00	1.33	0.06	-0.01	0.12
Chaos (work pace)	1.690	1.179	2.423	0.004	1.19	1.05	1.36	0.07	0.02	0.12
EMR Frustrating	1.156	.805	1.662	0.431	1.04	0.93	1.18	0.01	-0.02	0.06
Interruptions	.759	.494	1.166	0.209	0.914	0.796	1.04	-0.03	-0.08	0.01
Lack of sleep	2.577	1.758	3.780	0.000	1.39	1.21	1.59	0.14	0.08	0.20
Positive Relationships with staff	1.112	.632	1.956	0.712	1.03	0.859	1.24	0.01	-0.06	0.08
Peer support	.582	.271	1.252	0.167	0.842	0.667	1.06	-0.07	-0.18	0.03
Recognition by program	.606	.410	.895	0.012	0.846	0.742	0.965	-0.07	-0.12	-0.01
Constant	1.090	.301	3.944	0.895						
Organization Variance	.108	.007	1.60							
		McKelvey&Zavoina-Pseudo-R2 = 0.55								

Percent burnout variance explained by the complete model = 55%. *AOR* adjusted Odds Ratio, *ARR* adjusted Relative Risk, *ARD* absolute risk difference, *EMR* Electronic Medical Record

^*^Odds Ratios adjusted for gender, year of training, and clustering of residents within institutions

#### Weekly time spent on different activities

In describing time spent, 18% had 6 h/week or more of home EMR time. In the average 63.6 h work week, there were 24.5 h direct patient care, 21 h indirect care, 7.2 h administrative work, 5.3 h teaching, and 3.2 h research. There was considerable variability in EMR time, with 305 residents (53.4% of 571 responding) spending 20 h per week or less on indirect care activities, 137 (24.0%) spending 20–30 h per week, 71 (12.4%) spending 30–40 h per week, and 58 (10.2%) spending > 40 h per week on indirect care. Thus 47% spent more than 20 h per week on the EMR, while 53% spent less than 20 h per week.

#### Qualitative findings

There were 497 comments for analysis, once blank and N/A responses were removed. Major themes related to 1) individual-level activities, 2) residency-specific issues or 3) system-level challenges.

*Individual-level activities* encompassed self-care practices, including adequate sleep, healthy meals, exercise, and time spent with family and friends. Respondents reported difficulty finding balance between work and home life, with some preferring to focus on wellness away from work. A female PGY 2 expressed that she had ‘no time to make her doctor’s appointments, much less find time to exercise’.

*Themes related to residency programs* included requests for structured curricula, a desire for more program director/attending support, need for control over one’s schedule, and acknowledging the difficult learning curve generated by yearly transitions. A male PGY 1 noted, “Major stresses include being new on my teams, learning the systems, and better understanding my role.”

*System challenges* included excessive workload, insufficient resources and staff, lack of leader support, and disproportionate time spent on documentation. One female PGY 2 related “I have been working too many unsustainable hours… I come home and I have even more documentation… none of that [documentation] time… is even counted in my working hours. I am completely drained, feeling under-appreciated and very burned out.” The experience of working within broken systems was expressed by one female PGY 3: “…these problems are not unique to (our) residency…: residents in the US are learning and training in a broken healthcare system.”

These findings, with qualitative data enhancing the list of contributing variables, allowed construction of a *conceptual model* (Supplemental Fig. 3A) illustrating work conditions associated with residents’ burnout. While most variables were tested in this study (in bold in the Figure), some seen in prior studies await future investigation.

## Discussion

Our national study in 36 different types of residency programs with current data in 1118 residents provides the substrate to answer a recently posed question concerning resident wellness after the pandemic: “How does healing occur?” [27] We found burnout was prevalent (42%), though somewhat less frequent than pre-pandemic (45% [7]) and less frequent than in currently practicing physicians (48% [5], and > 50% [28, 29]). Effective teamwork, peer support and staff support were high (endorsed by 87–94% of residents), and may have protected against higher burnout. Values alignment with leadership was strongly associated with lower burnout. Meanwhile, burnout was accompanied by lack of work control, sleep impairment, and chaotic environments. While recognition by programs related to lower burnout, it was only present in 2/3 of residents; this may represent an opportunity for improvement if confirmed in further investigations. Work conditions in females were less favorable in most areas, with all work environment subscales substantively lower (poorer) for females. PGY 1’s had the most favorable scores among PGY 1’s, 2’s and 3’s, and PGY 2’s had poorer scores in several areas with less supportive work environments. Finally, EMR time varied considerably, and was a concern in open-ended comments. Due to convenience sampling and allowing for multiple comparisons to identify potential remediable worklife factors, these findings should be viewed as exploratory; yet they also paint a picture of worklife in residency with specific areas for improvement and some areas of success (peer support, values alignment and teamwork) to maintain and build upon.

Our data close gaps in the literature by 1) presenting *national findings* for worklife factors related to burnout in a large and diverse sample of residents and residencies, 2) describing the *prevalence of key aspects of favorable work cultures* and community building, including peer support, teamwork and staff relationships, 3) highlighting the need to learn more about the details of the potential impact *of sleep impairment*, 4) noting *recognition by program* as a potential means to reduce burnout, 5) demonstrating persistent and seemingly worsening *findings of gender differences* in burnout and 6) describing contributors to *less supportive environments among PGY 2’s.*The literature has shown indicators of burnout within medical residents [7–9, 30], and a wide range of burnout prevalence (from 25–75%). Dyrbye’s national studies published in 2018 [7] demonstrate higher rates of burnout in female and PGY-2 residents, but little information on differences in work conditions. Rodrigues, in 2018 [9] demonstrated overall burnout rates of 35%. While Nene’s recent blogpost [31] resonates with Ishak’s list of proposed system changes [8], including workload reduction, mentoring, and work family balance, and individual interventions such as stress management and meditation, the impact of these strategies remains to be tested.

There are reaffirming findings in our data of *what has occurred to build a community around residents*, including a high prevalence of peer support, clinical staff support and teamwork. In some subgroups, these were strikingly high (e.g. good to excellent teamwork endorsed by 98% of PGY 1’s). With strong literature evidence for these workplace attributes [24], the worklife aspects presented here comprise a foundation for measurement and monitoring to allow program directors to determine effectiveness of their support systems.

Regression analyses determined remediable *factors that are related to burnout*, including sleep impairment, lack of work control and fast paced, chaotic environments. While burnout has diminished with duty hour restrictions [32, 33], it has not been eliminated; sleep impairment was described by a third of residents and, with confirmation in future studies and more details of aspects of sleep impairment that are most prevalent, may represent an opportunity for improvement, with customized schedules (e.g., with jeopardy call back-up [14]) to address sleep challenges in real time. Work control was a major factor for burnout in the early pandemic [1], and work overload currently contributes to burnout across the healthcare workforce [34]; customizing workloads to individuals’ work capacity could be tested as a means to reduce burnout and distress. Finally, chaos (fast-paced, hectic workplaces) has been a challenge for physicians [26], yet few programs have developed metrics to monitor and adjust workplaces (e.g. using human-centered design) for more calm and reasonable workplaces. We propose these factors (sleep, work control and chaos) as part of a program’s Key Performance Indicator (KPI) worklife dashboard.

While *gender differences*have long been known, with higher burnout rates among female physicians in practice and academia [35, 36], their prevalence in residents has recently been noted [7, 37], though described in mainly small, localized studies, or with only modest differences (7.6% risk difference in 2018) [7]. Our findings suggest an absolute burnout increase of over 20% in females, with most worklife items showing poorer scores among female residents, including control, chaos, home EMR use, program recognition and sleep impairment. Other potential contributors include parental responsibilities, harassment and discrimination [38], gendered expectations for listening [39], excess “invisible work” in female physicians [40] and low autonomy [41]. Strategies to reduce gender differences [39] include improving understanding of lived experiences, creating interventions to value invisible work, addressing EMR inequities [42, 43], and providing greater control of workload to mesh with off-duty responsibilities. With monitoring and transparency, gender inequities can, we believe, be reduced and, eventually, eliminated.

We found an *excess of burnout in those preferring not to identify (PNTI) gender or race*, with burnout rates of approximately 56% vs 42% in others. Prior studies demonstrated high burnout among LGBTQ students compared with heterosexual students [44]. Thus, surveys may be missing input from high-stress gender and racial groups; additional efforts are warranted to determine how to best reach out to these groups of trainees.

An unanticipated finding was the low rate of burnout among PGY 1’s and *challenging work conditions of PGY 2’s*. Norvell [37] suggests a program for residents transitioning from PGY-1 to PGY 2; others propose a PGY 2 curriculum. In internal medicine programs, the stress of fellowship applications is often highest during the PGY 2 year. Worklife factors meriting attention include home EMR use, sleep impairment, teamwork and program recognition. Supportive work environment subscales were lowest among PGY 2’s (small to moderate Effect Size vs PGY 1’s, *p* < 0.001). Thus, attention to the PGY 2 year seems warranted.

*Qualitative findings* demonstrated 3 themes: individual-level factors, residency-specific aspects, and system-level problems. Self-care needs included available time to rest/sleep, exercise, connect with friends, balance work with family, and take care of one’s own health (e.g. doctor’s appointments). Meditation, mentioned by only a few respondents, was related to low burnout in one randomized trial [45] while exercise led to burnout reductions in a pre-post trial [46]. In the current study, residents proposed areas for change, including better curricula, control of schedule, mitigation of long hours, support with year-to-year transitions, workload adjustment, program leader support, and more explicitly being valued.

These factors, along with pandemic-specific frustrations such as lack of support staff, and the quantitative findings noted above, comprise a conceptual model explaining resident burnout (Supplemental Fig. 3A). The 55% of variance in burnout explained by quantitatively measured variables in this model is among the highest in reported physician burnout models.

For *interventions*, Vijay and Yancy [27] propose “changing the vernacular” of what is a good doctor during training from one always present, to one with good team participation, work-life balance and valuing life moments inside and outside of work. They describe residents’ appreciation of the Hopkins Bayview Aliki Service, with fewer patients per resident, attention to social determinants of health, and deeper connections with patients and community. They note a need for time to recover from traumatic events, highlighting recovery programs from recent traumas. Our methods provide a useful means of supporting these suggestions, with a focus on measurement and benchmarking of worklife factors, alleviating gender differences, improving PGY 2 work conditions, addressing EMR excess (e.g. with scribes [47]), assessing workload and upgrading parental leave policies [31, 48]. Recognizing residents’ efforts, straightforward and inexpensive, could quickly address satisfaction and sense of community in women and PGY 2s.

Our work has several *limitations and strengths*. While ours is a convenience sample, it is a national sample including measurement of worklife among residents and fellows in dozens of program types for which there are few precedents. The 32% response rate, though less than optimal, exceeds the standard 7–20% response rates of national physician surveys [29, 49]; we also have little if any information which could allow us to estimate the degree of non-response bias, and some organizational response rates were estimated or inaccurate. While the burnout item is validated against mainly the emotional exhaustion subscale in the Maslach instrument, other Mini Z items correlate with exhaustion and depersonalization [19]. Survey timing may have been different among PGY 1’s, 2’s and 3’s, accounting in part for some differences. Furthermore, worklife and wellness may vary considerably throughout the year; this variation is not accounted for by our analyses. As for strengths, survey items and the Mini ReZ are well validated [10, 11], and mixed methods provide confirmation and enhancement of factors facing residents; furthermore, the data are reasonably current, as of late April 2023. This lends both urgency and temporal validity to the findings.

## Conclusions/implications

Residents perceive strong support by staff, peers and clinical teams. However, burnout rates still exceed 40% nationally, and are higher among females and PGY 2’s. Addressing workload, EMR use, sleep impairment and chaotic environments, as well as providing clear recognition of resident efforts, are evidence-based strategies to pursue for burnout reduction. Future studies could measure the impact of interventions, time spent on varied aspects of work and care, and mechanisms to better reach those not identifying race or gender.

### Supplementary Information

**Supplementary Material 1.**

**Supplementary Material 2.**

**Supplementary Material 3.**

**Supplementary Material 4.**

**Supplementary Material 5.**

**Supplementary Material 6.**

## Data Availability

The data that support the findings of this study are provided from the American Medical Association. Restrictions apply to the availability of these data, which were obtained through individual data use agreements from engaged residency programs, and thus are not publicly available. An aggregate, de-identified data set can be obtained from the corresponding author upon reasonable request.

## Abbreviations

AMA: American Medical Association
aOR: Adjusted Odds Ratio
EMR: Electronic Medical Record.
ES: Effect Size
FDR: False Discovery Rate
PGY: Post Graduate Year
PNTI: Prefer not to indicate gender or race
MBI: Maslach Burnout Inventory
