# Supplementary material for: Resident worklife and wellness through the late phase of the pandemic: a mixed methods national survey study
Source: BMC Med Educ. 2024 May 2;24:484. doi: 10.1186/s12909-024-05480-5 (PMC11064291; doi:10.1186/s12909-024-05480-5)
Supplement: Supplementary file 1 — Supplementary Material 1. [file 12909_2024_5480_MOESM1_ESM.docx]

Technical appendix:

For the Mini ReZ, a summary score of 75 (5 x 15, range 15-75) is created, consisting of three 5 item subscales: 1) supportive work environment (range 5-25), 2) work pace/EMR stress (range 5-25) and 3) resident specific factors (sleep, interruptions, peer and staff support, and program recognition, range 5-25). In a 5 choice item, burnout was considered as “present” if any of the first 3 choices were endorsed, all of which included the word burnout. This item has been validated against the Maslach Burnout Inventory (MBI) Emotional Exhaustion subscale ^12^ and has been used for 20 years since the Physician Worklife Study ^17^ . In more recent studies ^18^, the prevalence of burnout (43%) in a national sample of practicing physicians from the AMA Masterfile using this single item was comparable to burnout prevalence measured using the full MBI, although in other studies the prevalence of burnout has been somewhat lower using the single item measure^19^.
