# Supplementary material for: Resident worklife and wellness through the late phase of the pandemic: a mixed methods national survey study
Source: BMC Med Educ. 2024 May 2;24:484. doi: 10.1186/s12909-024-05480-5 (PMC11064291; doi:10.1186/s12909-024-05480-5)
Supplement: Supplementary file 2 — Supplementary Material 2. [file 12909_2024_5480_MOESM2_ESM.docx]

Supplemental Table 1A. Distribution of residency and fellowship programs

|  | **Total** | **PGY1** | **PGY2** | **PGY3** | **PGY4** | **PGY5** | **Fellow** | **Missing** |
| --- | --- | --- | --- | --- | --- | --- | --- | --- |
| **Total** | **1118** | **266** | **269** | **234** | **130** | **50** | **124** | **45** |
| **Missing** | **271** | 58 | 48 | 60 | 34 | 6 | 20 | 45 |
| **Internal Medicine, General - Primary Care** | **128** | 56 | 40 | 30 | 1 |  | 1 |  |
| **Family Medicine** | **96** | 36 | 37 | 23 |  |  |  |  |
| **Emergency Medicine** | **70** | 20 | 20 | 20 | 5 | 1 | 4 |  |
| **Orthopedic Surgery** | **49** | 10 | 9 | 8 | 10 | 7 | 5 |  |
| **Pediatrics** | **47** | 17 | 19 | 10 |  |  | 1 |  |
| **Surgery, General** | **45** | 9 | 9 | 9 | 8 | 8 | 2 |  |
| **Obstetrics And Gynecology** | **41** | 9 | 5 | 8 | 11 | 1 | 7 |  |
| **Physical Medicine And Rehabilitation** | **34** | 9 | 9 | 7 | 8 | 1 |  |  |
| **Hospitalist** | **31** | 5 | 13 | 12 | 1 |  |  |  |
| **Psychiatry** | **31** | 6 | 3 | 7 | 8 | 2 | 5 |  |
| **Radiology** | **31** |  | 11 | 10 | 3 | 5 | 2 |  |
| **Neurology** | **26** | 4 | 8 | 4 | 8 | 1 | 1 |  |
| **Gastroenterology** | **21** |  | 1 |  | 3 | 2 | 15 |  |
| **Other Non-Surgery Related Specialty** | **21** | 3 | 4 | 2 | 2 | 3 | 7 |  |
| **Otolaryngology** | **18** | 2 | 3 | 2 | 7 | 4 |  |  |
| **Pathology** | **15** | 4 | 3 | 3 | 2 |  | 3 |  |
| **Podiatry** | **15** | 7 | 4 | 4 |  |  |  |  |
| **Hematology/Oncology** | **11** |  | 1 |  | 1 |  | 9 |  |
| **Ophthalmology** | **11** |  | 3 | 4 | 2 |  | 2 |  |
| **Critical Care Medicine** | **10** |  | 1 |  | 1 |  | 8 |  |
| **Cardiovascular Diseases** | **9** |  | 1 |  | 3 |  | 5 |  |
| **Plastic Surgery** | **9** | 2 | 1 | 2 | 2 | 2 |  |  |
| **Neurological Surgery** | **8** |  | 1 |  | 1 | 2 | 4 |  |
| **Dentistry/Oral Surgery** | **7** | 4 |  | 1 | 2 |  |  |  |
| **Dermatology** | **7** |  | 1 | 3 | 1 |  | 2 |  |
| **N/A** | **7** |  | 4 | 1 |  |  | 2 |  |
| **Nephrology** | **7** |  |  |  | 2 | 2 | 3 |  |
| **Other Surgery-Related Specialty** | **7** | 2 | 2 | 1 |  |  | 2 |  |
| **Anesthesiology** | **6** | 1 | 4 |  | 1 |  |  |  |
| **Infectious Disease** | **5** |  |  |  | 1 |  | 4 |  |
| **Vascular Surgery** | **5** |  | 1 |  |  | 1 | 3 |  |
| **Radiation Oncology** | **4** |  |  | 2 | 1 | 1 |  |  |
| **Urological Surgery** | **4** | 1 | 1 | 1 |  | 1 |  |  |
| **General Practice** | **3** | 1 | 1 |  |  |  | 1 |  |
| **Palliative Care** | **3** |  |  |  |  |  | 3 |  |
| **Pulmonary Disease** | **3** |  | 1 |  |  |  | 2 |  |
| **Rheumatology** | **2** |  |  |  | 1 |  | 1 |  |
