# Supplementary material for: Resident worklife and wellness through the late phase of the pandemic: a mixed methods national survey study
Source: BMC Med Educ. 2024 May 2;24:484. doi: 10.1186/s12909-024-05480-5 (PMC11064291; doi:10.1186/s12909-024-05480-5)
Supplement: Supplementary file 3 — Supplementary Material 3. [file 12909_2024_5480_MOESM3_ESM.docx]

Supplemental Table 2A. Full regression model including adjusting covariates of gender and Post Graduate Year.

|  |  |  |  |  |  |  |
| --- | --- | --- | --- | --- | --- | --- |
| Burnout | Odds ratio | Std. err. | z | P>\|z\| | 95% confidence intervals |  |
|  |  |  |  |  |  |  |
| Satisfaction | .415 | .116 | -3.14 | 0.002 | .240 | .719 |
| Values alignment | .428 | .101 | -3.56 | 0.000 | .268 | .682 |
| Teamwork efficiency | .552 | .282 | -1.16 | 0.245 | .203 | 1.50 |
| Lack of work control | 2.03 | .488 | 2.96 | 0.003 | 1.27 | 3.25 |
| High stress | 4.47 | .828 | 8.09 | 0.000 | 3.11 | 6.43 |
| EMR outside of work | .956 | .198 | -0.21 | 0.832 | .636 | 1.43 |
| Documentation time pressure | 1.55 | .342 | 1.98 | 0.047 | 1.00 | 2.39 |
| Work atmosphere (chaos) | 1.69 | .310 | 2.86 | 0.004 | 1.17 | 2.42 |
| EMR Frustrating | 1.15 | .213 | 0.79 | 0.431 | .805 | 1.66 |
| Interruptions | .759 | .166 | -1.26 | 0.209 | .494 | 1.16 |
| Lack of sleep | 2.57 | .503 | 4.85 | 0.000 | 1.75 | 3.78 |
| Support staff relationships | 1.11 | .320 | 0.37 | 0.712 | .632 | 1.95 |
| Peer support | .582 | .227 | -1.38 | 0.167 | .271 | 1.25 |
| Program recognition | .606 | .120 | -2.51 | 0.012 | .410 | .895 |
|  |  |  |  |  |  |  |
| Gender |  |  |  |  |  |  |
| Female | 2.04 | .358 | 4.06 | 0.000 | 1.44 | 2.87 |
| PNTI | 1.46 | .587 | 0.94 | 0.345 | .664 | 3.21 |
| Missing | .827 | .868 | -0.18 | 0.857 | .105 | 6.47 |
|  |  |  |  |  |  |  |
| Post Graduate Year (PGY) |  |  |  |  |  |  |
| PGY2 | 1.82 | .427 | 2.55 | 0.011 | 1.14 | 2.88 |
| PGY3 | 2.16 | .539 | 3.12 | 0.002 | 1.33 | 3.53 |
| PGY4+PGY5+fellow | 1.37 | .335 | 1.31 | 0.190 | .853 | 2.21 |
|  |  |  |  |  |  |  |
| _cons | 1.09 | .715 | 0.13 | 0.895 | .301 | 3.94 |
|  |  |  |  |  |  |  |
|  |  |  |  |  |  |  |

PNTI = prefer not to identify. Reference group for gender = male; reference group for PGY is PGY1.
