# Supplementary material for: Resident worklife and wellness through the late phase of the pandemic: a mixed methods national survey study
Source: BMC Med Educ. 2024 May 2;24:484. doi: 10.1186/s12909-024-05480-5 (PMC11064291; doi:10.1186/s12909-024-05480-5)
Supplement: Supplementary file 4 — Supplementary Material 4. [file 12909_2024_5480_MOESM4_ESM.docx]

Supplemental Figure 1A. Mini ReZ 2.0 version with scoring

**Mini ReZ survey (for residents and fellows)**

**For questions 1-10, Please indicate, based on your experience for the majority of days OVER THE PAST MONTH:**

**1. I have been satisfied with my residency/fellowship program:** *[Scoring: Responses 4-5 = satisfied]*

1=Strongly disagree 2=Disagree 3=Neither agree nor disagree 4=Agree 5=Agree Strongly

**2. Using your own definition of "burnout", please choose one of the answers below:** *[Scoring: responses 1-3 = burnout]*

1=I feel completely burned out. I am at the point where I may need to seek help.

2=The symptoms of burnout that I'm experiencing won't go away. I think about work frustrations a lot.

3=I am beginning to burn out and have one or more symptoms of burnout, e.g. emotional exhaustion

4=I am under stress, and don’t always have as much energy as I did, but I don’t feel burned out.

5=I enjoy my work. I have no symptoms of burnout.

**3. My professional values are well aligned with those of my program leaders:** *[Scoring: Responses 4-5 = high values alignment]*

1=Strongly disagree 2=Disagree 3=Neither agree nor disagree 4=Agree 5=Agree Strongly

**4. The efficiency of my team has been:** *[Scoring: Responses 3-5 = good teamwork]*

1=Poor 2=Marginal 3=Satisfactory 4=Good 5=Optimal

**5. My control over my workload has been:** *[Scoring: Responses 1-2 = poor workload control]*

1 = Poor 2 = Marginal 3 = Satisfactory 4=Good 5=Optimal

**6. I have felt a great deal of stress because of my job:** *[Scoring: Responses 1-2 = high stress]*

1=Agree strongly 2=Agree 3=Neither agree nor disagree 4=Disagree 5=Strongly disagree

**7. The amount of time I have spent on the electronic health record (EHR) after hours is:** *[Scoring: Responses 1-2 = too much EMR work at home]*

1=Excessive 2=Moderately high 3=Satisfactory 4=Modest 5=Minimal/none

**8. The amount of time I spend on documentation is...** *[Scoring: Responses 1-2 = poor documentation]*

1=Excessive 2=Moderately high 3=Satisfactory 4=Modest 5=Minimal/none

**9. Which selection best describes the atmosphere in your work area (for the majority of the past month)?** *[Scoring: Responses 1-2 = chaos]*

1= Hectic, chaotic 3= Busy, but reasonable 5=Calm

**10. The electronic health record (EHR) added to the frustration of my day:** *[Scoring: Responses 1-2 = frustration]*

1=Agree strongly 2=Agree 3=Neither agree nor disagree 4=Disagree 5=Strongly disagree

**For questions 11-12, How have the following items diminished your job satisfaction over the past month?**

**11. Work interruptions (e.g. pages greater than expected, etc.):** *[Scoring: Responses 1-2 = presence of work interruptions]*

1=A lot 2=A moderate amount 3=Somewhat 4=A little 5=Not at all

**12. Lack of sleep:** *[Scoring: Responses 1-2 = presence of lack of sleep]*

1=A lot 2=A moderate amount 3=Somewhat 4=A little 5=Not at all

**For questions 13-15, How have the following items improved your job satisfaction over the past month?**

**13. Positive relationships with clinical support staff:** *[Scoring: Responses 3-5= presence of positive relationships]*

1=Not at all 2=A little 3=Somewhat 4=A moderate amount 5=A lot

**14. Support by peers:** *[Scoring: Responses 3-5= feels supported by peer]*

1=Not at all 2=A little 3=Somewhat 4=A moderate amount 5=A lot

**15. Recognition by my department:** *[Scoring: Responses 3-5= feels recognized by department]*

1=Not at all 2=A little 3=Somewhat 4=A moderate amount 5=A lot

**16. Tell us more about your current stresses and ideas you have for minimizing them:**

Scoring your Mini ReZ: add the numbered responses from questions 1-15. Range 15-75 (>= 60 is a positive learning environment).

Subscale 1 – Supportive Work Environment: add the numbered responses to questions 1-5. Range 5-25 (>=20 is a highly supportive work environment)

Subscale 2 – Work pace and EMR Stress: add the numbered responses to questions 6-10. Range 5-25 (>=20 is an environment with good pace and manageable EMR stress)

Subscale 3 – Resident Experience: add the numbered responses to questions 11-15. Range 5-25 (>=20 is a positive and healthy resident experience)

**For group scoring of individual items:**

1. Satisfaction - responses 4 and 5 = % satisfied

2. Burnout - responses 1, 2 and 3 = % burned out

3. Values alignment - responses 4 and 5 = % values aligned

4. Teamwork - responses 3, 4 and 5 = % good teamwork

5. Control of work - responses 1 and 2 = % poor work control

6. High stress - responses 1 and 2 = % high stress

7. EHR after hours - responses 1 and 2 = % excess EHR time at home

8. Time pressure for documentation - responses 1 and 2 = % poor documentation

9. Chaos - responses 1 and 2 = % with chaotic environments

10. EHR frustration - responses 1 and 2 = % with EHR frustrations

11. Interruptions - responses 1 and 2 = % with frequent interruptions

12. Sleep impairment - responses 1 and 2 = % with moderate or higher sleep impairment

13. Relationships with support staff - responses 3, 4 and 5 = % with good support staff relationships

14. Peer support - responses 3, 4 or 5 = % with good peer support

15. Program recognition - responses 3, 4 or 5 = % with good program recognition

**The Mini Z was developed by Dr. Mark Linzer and team at Hennepin Healthcare, Minneapolis MN. Mini Z survey tools can be used for research, program evaluation and education without restriction. Permission for commercial or revenue-generating applications of the Mini Z must be obtained from Mark Linzer or Hennepin Healthcare Institute for Professional Worklife prior to use: www.professionalworklife.com.*
