# Supplementary material for: Resident worklife and wellness through the late phase of the pandemic: a mixed methods national survey study
Source: BMC Med Educ. 2024 May 2;24:484. doi: 10.1186/s12909-024-05480-5 (PMC11064291; doi:10.1186/s12909-024-05480-5)
Supplement: Supplementary file 5 — Supplementary Material 5. [file 12909_2024_5480_MOESM5_ESM.docx]

Supplemental Figure 2A. Forest plot of gender and PGY year differences in worklife subscales for 1118 residents in national resident survey. Subscale 1 = supportive work environment; subscale 2 = work pace/EMR stress; subscale 3 = resident specific subscale (including interruptions, sleep impairment, recognition by program, staff relationships and peer support).


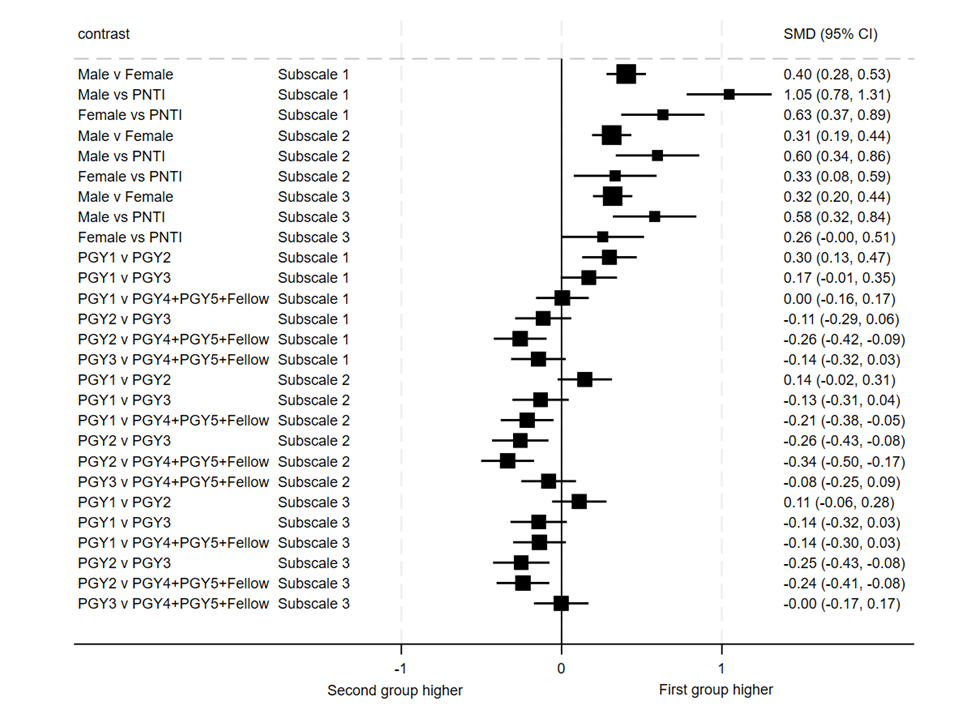


SMD = standardized mean difference (0.2 small, 0.5 moderate, > 0.8 = large). Subscale 1 = supportive environment; subscale 2 = work pace/EMR stress; subscale 3 = resident-specific subscale (sleep, interruptions, peer and staff support, program recognition). EMR = Electronic Medical Record. PNTI = Prefer not to identify gender.
