# Supplementary material for: Resident worklife and wellness through the late phase of the pandemic: a mixed methods national survey study
Source: BMC Med Educ. 2024 May 2;24:484. doi: 10.1186/s12909-024-05480-5 (PMC11064291; doi:10.1186/s12909-024-05480-5)
Supplement: Supplementary file 6 — Supplementary Material 6. [file 12909_2024_5480_MOESM6_ESM.docx]

Supplemental Figure 3A. Conceptual model of burnout during residency from national resident survey July 2022 to April 2023 (variables addressed in current study in bold).

| *Background variables* | *Proposed Mediators* | *Intermediate Outcomes* | *Final Outcomes* |
| --- | --- | --- | --- |
| **Gender***  **Race and Ethnicity***  Program type  **Year of training***  **Chaos***  Administrative work  **EMR***  **Teamwork***  **Values aligne**d* | **Self-care**  Feeling valued  **Work control***  Work family balance  Home support  **Sleep impairment***  **Program recognition***  **Leader support** | **Satisfaction***  **Stress***  Depression | **Burnout**  Intent to leave |

Percent burnout variance explained by asterisked variables in the model = 55%.
